# Fatty Acids Composition of Blood Cell Membranes and Peripheral Inflammation in the PREDIMED Study: A Cross-Sectional Analysis

**DOI:** 10.3390/nu11030576

**Published:** 2019-03-07

**Authors:** Jananee Muralidharan, Christopher Papandreou, Aleix Sala-Vila, Nuria Rosique-Esteban, Montserrat Fitó, Ramon Estruch, Miguel Angel Martínez-González, Dolores Corella, Emilio Ros, Cristina Razquín, Olga Castañer, Jordi Salas-Salvadó, Monica Bulló

**Affiliations:** 1 Department of Biochemistry and Biotechnology, Human Nutrition Unit, IISPV, Hospital Universitari Sant Joan de Reus, Rovira i Virgili University, 43003 Reus, Spain; jananee.muralidharan@estudiants.urv.cat (J.M.); papchris10@gmail.com (C.P.); nuria.rosique@urv.cat (N.R.-E.); 2 CIBER de Fisiopatología de la Obesidad y la Nutrición (CIBEROBN), Instituto de Salud Carlos III, 28029 Madrid, Spain; asala@clinic.cat (A.S.-V.); mfito@imim.es (M.F.); restruch@clinic.ub.es (R.E.); mamartinez@unav.es (M.A.M.-G.); dolores.corella@uv.es (D.C.); eros@clinic.ub.es (E.R.); crazquin@unav.es (C.R.); ocastaner@imim.es (O.C.); 3 Lipid Clinic, Department of Endocrinology and Nutrition, Institut d’Investigacions Biomediques August Pi Sunyer (IDIBAPS), Hospital Clinic, University of Barcelona, 08036 Barcelona, Spain; 4 Cardiovascular Risk and Nutrition (Regicor Study Group), Hospital del Mar Research Institute (IMIM), 08003 Barcelona, Spain; 5 Department of Internal Medicine, Hospital Clínic, IDIBAPS August Pi i Sunyer Biomedical Research Institute, University of Barcelona, 08036 Barcelona, Spain; 6 Department of Preventive Medicine and Public Health, IdiSNA, Navarra Institute for Health Research, University of Navarra, 31008 Pamplona, Spain; 7 Department of Preventive Medicine, University of Valencia, 46010 Valencia, Spain

**Keywords:** inflammation, fatty acids, cell membranes

## Abstract

There is limited evidence from epidemiological studies for the inflammatory or anti-inflammatory properties of fatty acids in blood cell membranes. Therefore, this study examined associations between baseline (*n* = 282) and 1-year (*n* = 143) changes in the levels of fatty acids in blood cell membranes with circulating inflammatory markers in older adults at high cardiovascular risk. The data for this cross-sectional analysis was obtained from a case-control study within the PREDIMED study. Linear regression with elastic net penalty was applied to test associations between measured fatty acids and inflammatory markers. Several fatty acids were associated with interferon-γ (IFNγ) and interleukins (ILs) IL-6, IL-8, and IL-10 at baseline and additionally also with IL-1b at 1 year. Omega-6 fatty acids were consistently positively associated with pro-inflammatory IL-6 and IL-8 at baseline. Omega-3 fatty acids including C20:5n3 and C18:3n3 were negatively associated with IFN-γ at 1 year. It is interesting to note that the cis and trans forms of C16:1n7 at 1 year were oppositely associated with the inflammatory markers. C16:1n7trans was negatively associated with IFN-γ, IL-6, IL-8, IL-10, and IL-1b, whereas C16:1n7cis was positively associated with IL-1b. This study adds to the growing body of evidence suggesting potential differences in inflammatory or anti-inflammatory properties of fatty acids in blood cell membranes.

## 1. Introduction

Inflammation plays a key role in a wide range of common chronic diseases, including type 2 diabetes (T2D), cardiovascular disease (CVD), neurodegenerative-related disorders, and cancer, and several cytokines have been implicated in the development of these diseases [1].

It is well established that the polyunsaturated fatty acid (PUFA) content of phospholipids influences inflammation. For example, omega-3 fatty acids and especially both eicosapentaenoic acid (EPA) and docosahexaenoic acid (DHA) are thought to have anti-inflammatory properties [2] compared to arachidonic acid, which is reported to be a precursor of many inflammatory eicosanoids. More recently, several epidemiological studies have also demonstrated that certain circulating saturated fatty acids (SFAs) might exacerbate inflammation [3]. However, the involvement of SFAs in inflammation may vary, depending on the type of SFA (i.e., chain length). Further research on inflammation and fatty acids expanded beyond PUFAs and SFAs, including monounsaturated fatty acids (MUFAs), as their plasma levels were recently negatively associated with different inflammatory markers such as interleukin (IL)-6, tumor necrosis factor (TNF alpha), IL-1b, and IL-8 [3].

Fatty acids are incorporated into the phospholipids of the inflammatory cell membranes, thus influencing inflammatory processes via a variety of mechanisms, acting via cell surface and intracellular receptors that control inflammatory cell signaling and gene expression [2]. In fact, the inflammatory response can be exacerbated or inhibited depending on the fatty acid composition of cell membranes.

No previous epidemiologic study has examined the association between the fatty acid composition of blood cell membranes and peripheral inflammation using repeated measurements of fatty acids to capture the dynamics of fatty composition changes. Therefore, the aim of the present cross-sectional study nested in the framework of the PREDIMED trial was to examine associations of baseline and 1-year changes in the levels of fatty acids of blood cell membranes with inflammatory markers in a Mediterranean population at high cardiovascular risk.

## 2. Methods

### 2.1. Study Design and Population

This is a cross-sectional analysis using baseline and 1-year data of a case-control study within the PREDIMED study. The PREDIMED study is a large multicenter trial for the prevention of cardiovascular disease (CVD) conducted in an elderly Spanish population at high cardiovascular risk. The participants were allocated to three dietary intervention groups: (1) Mediterranean diet supplemented with extra virgin olive oil (MedDiet+EVOO); (2) Mediterranean diet supplemented with mixed nuts (MedDiet+nuts); and (3) Advice to adhere to a low-fat diet (Control). Eligible participants were men (55–80 years) and women (60–80 years) free from CVD at baseline who were previously diagnosed with either T2D or three or more cardiovascular risk factors: current smoking, hypertension (blood pressure >140/90 mmHg or treatment with antihypertensive drugs), low density lipoprotein (LDL)-cholesterol >160 mg/dL (or treatment with hypolipidemic drugs), high density lipoprotein (HDL)-cholesterol (≤50 mg/dL in women or ≤40 mg/dL in men), overweight/obesity (body mass index (BMI) ≥ 25 kg/m^2^), or having a family history of premature coronary heart disease. The exclusion criteria were history of CVD, any severe chronic illness or low predicted likelihood of changing dietary habits according to the stages of change model, among others [4]. All participants provided written informed consent. The PREDIMED study was approved by the institutional review boards of all recruiting centers according to the ethical standards of the Declaration of Helsinki. The trial was registered at the International Standard Randomized Controlled Trial (ISRCT: http://www.isrctn.com/ISRCTN89898870) with number 89898870 and registration date of 24 July 2014.

In the PREDIMED study, a total of 280 cases of coronary heart disease were reported. In order to achieve a case-to-control ratio of 1:2, a total of 840 participants was considered for the study (280 cases and 560 controls). Of these 840 participants, 408 had available buffy coat samples for fatty acid analysis and 301 participants had available serum samples for inflammatory marker analysis. Therefore, a total of 282 participants (105 cases and 177 controls) had data for both fatty acid and inflammatory markers, while 143 participants (57 cases and 86 controls) were included in the 1-year change analyses.

### 2.2. Biological Samples Collection and Storage

Blood samples were collected after overnight fasting at baseline and at 1-year follow-up. Whole blood was obtained in vacutainers for further processing of serum. Serum samples were aliquoted and stored at −80 °C until further processing. Buffy coat was separated from the fresh blood samples and frozen at −80 °C until further processing.

### 2.3. Inflammatory Markers in Serum

Serum inflammatory markers including interferon-gamma (IFNγ) and interleukins (ILs) IL-1b, IL-6, IL-8, and IL-10 were determined using a MILLIPLEX MAP Plex Kit (Merck Millipore, Billerica, MA, USA). The ELISA plate provided was washed with 200 µL of 1× wash buffer and shaken for 10 min at room temperature. Fifty microliters of provided standard control was added to their respective wells, followed by 50 µL of serum matrix (MXHSM-7) to the background wells. Twenty-five microliters of assay buffer was added to sample wells and 25 µL samples were added in duplicates. Finally, 25 µL of beads were added to all the wells and the plate was sealed with a plated sealer and incubated with agitation in a plate shaker overnight at 4 °C. After the overnight incubation, the plate was washed thrice with wash buffer and 50 µL of detection antibodies were added. After a 60-min incubation at room temperature, 50 µL of streptavidin-phycoerythrin was added to each well. This was further incubated for 30 min at room temperature followed by washing (three times). One hundred and fifty microliters of drive fluid was added to each well followed by a final incubation for 5 min at room temperature. The plate was then read on Luminex 200^TM^ (Luminex Corp., Austin, TX, USA) and xPONENT software version 3.1. The data was saved and analyzed using a five-parameter logistic model to determine the analyte concentrations.

### 2.4. Fatty Acid Composition in Blood Cell Membranes

The blood cell membrane fatty acid profile was assessed by gas-chromatography as described elsewhere [5]. To discard the hemoglobin and any remnant of serum lipids, diluted buffy coat (100 mL in 1400 mL of distilled water) was spun and the supernatant was discarded. The pellet (>99% Red blood cell (RBC) membranes) was dried, dissolved in 1 mL BF3 methanol solution, and heated; in order to hydrolyze and methylate glycerophospholipid fatty acids, 300 mL of n-hexane was added to this extract and fatty acid methyl esters were isolated. An aliquot of hexane layer was used after centrifugation and transferred into an automatic injector vial equipped with 300 mL of volume adapter. Agilent HP 7890 gas chromatograph (Agilent Technologies, Inc., Santa Clara, CA, USA) equipped with a 30 m × 0.25 μm × 0.25 mm SupraWAX-280 capillary column (Teknokroma, Barcelona, Spain), an autosampler, and a flame ionization detector were used to separate the fatty acid methyl esters.

The method used to quantify the fatty acids has been cross-validated against the method used in the original definition of the omega-3 index proposed by Harris and Von Schacky [5]. Total saturated fatty acids (SFAs) was calculated by summing the percentage of C14:0, C16:0, C18:0, C20:0, C22:0, and C24:0. Total monounsaturated fatty acids (MUFAs) were the sum of C16:1n7 cis and trans, C18:1n9 cis and trans, C20:1n9, and C24:1n9. Total n-6 polyunsaturated fatty acids (PUFAs) were the sum of C18:2n6, C20:2n6, C20:3n6, C20:4n6, C22:4n6, and C22:5n6. Long-chain n-3PUFA was the sum of C20:5n3, C22:6n-3, and C22:5n3. Very-long chain saturated fatty acids (VLCSFAs) was the sum of C20:0, C22:0, and C24:0.

### 2.5. Assessment of Other Variables

Lifestyle variables such as smoking status, medical history, and medication(s) used were determined at baseline by a 47-item questionnaire. Physical activity was assessed using a validated Spanish version of the Minnesota Leisure Time Physical Activity Questionnaire [6]. Waist circumference (WC) was measured midway between the lowest rib and the iliac crest using an anthropometric tape. Fasting plasma was used to determine participants’ triacylglycerol (TAG), total cholesterol, HDL-cholesterol, and LDL-cholesterol levels.

### 2.6. Statistical Analyses

Baseline characteristics of study participants were described as means and standard deviations (SDs). The amount of each fatty acid in blood cell membranes is expressed as a percentage of the total identified fatty acid. We applied a natural logarithmic transformation to approximate a normal distribution of fatty acid levels in blood cell membranes and serum inflammatory markers.

Due to the high dimensionality and collinear nature of the data, linear regression with elastic net penalty was implemented in the “glmnet” R package (alpha = 0.5) to examine associations between fatty acids and inflammatory markers. We also tested the associations between 1-year changes in fatty acids levels and 1-year changes in inflammatory markers. With respect to fatty acids and inflammatory markers, we first calculated the ratio between 1-year and baseline values and then normalized this ratio with the natural logarithmic transformation. Ten-fold cross-validation was performed to find the optimal value of the tuning parameter that resulted in a mean-squared error (MSE) within 1 SD of the minimum [7]. The values minMSE and minMSE + 1 SE were estimated using the argument s = “lambda.min” in the cv.glmnet function. Models were adjusted for age (years), sex (male or female), BMI (kg/m^2^), T2D (yes/no), smoking (never, current, former), and use of statins (yes/no). Additionally, we adjusted for intervention group in the 1-year change models. All analyses were performed using R statistical package 3.4.3 (www.r-project.org) (R Development Core Team, 2012).

## 3. Results

### 3.1. Participant Characteristics

Two hundred and eighty-two subjects (174 males and 108 females) were included in the analysis. The general characteristics of the study subjects are shown in Table 1. The mean age of participants was 67.9 years, and the mean ± standard deviation BMI was 29.4 ± 3.1. Overall, the observed prevalence was 55.8% for T2D and 68.2% for dyslipidemia, while 19.1% of participants were current smokers. The percentages of the total cell membrane fatty acid compositions for the study population at baseline are shown in Table 2. The percentages of the total cell membrane fatty acid compositions for both cases and controls are shown in Table S1 (see the supplementary materials). Table S2 shows the inflammatory markers measurement by case status. Spearman correlation coefficients between baseline and 1-year measures of fatty acids and inflammatory markers are shown in Tables S3 and S4, respectively.

### 3.2. Associations between Baseline Fatty Acids and Baseline Inflammatory Markers

Table 3 show selected fatty acids ranked from the highest to the lowest elastic net positive and negative regression coefficients for inflammatory markers. C20:1n9 negatively associated with IFN-γ, while other fatty acids including C14:0, C20:0, C16:1n7trans, and C18:3n3 were negatively associated with IL-6. C20:2n6 was positively associated with IL-6. Positive associations of several individual fatty acids (C20:3n6, C22:5n6) with IL-8 and negative associations of total MUFA and C14:0 with IL-8 were observed. No fatty acid was associated with IL-10 or IL-1b.

### 3.3. Associations between 1-Year Changes in Fatty Acids and 1-Year Changes in Inflammatory Markers Values

Table 4 shows selected fatty acids ranked from the highest to the lowest elastic net positive and negative regression coefficients for inflammatory markers. Negative regression coefficients with changes in IFN-γ were found for changes in C16:1n7trans and C24:1n9. Changes in the C16:1n7trans were also negatively associated with changes in IL-6. IL-8 was positively associated with C22:5n6 and negatively associated with C16:1n7trans and C18:1n9cis. Changes in two fatty acids (C14:0 and C16:1n7trans) were negatively associated, while C20:2n6 was positively associated with changes in IL-10. Changes in several fatty acids (C14:0, C16:1n7trans, C24:1n9, C18:3n3) were negatively associated, while only C16:1n7cis was positively associated with changes in IL-1b.

## 4. Discussion

In this cross-sectional study within a case-control substudy in the context of the PREDIMED trial, several fatty acids of blood cell membranes were associated with serum levels of different inflammatory markers in 282 subjects at high CVD risk. Consistently, 1-year changes in blood cell fatty acids levels were associated with changes in all the inflammatory markers analyzed. These results highly suggest that the fatty acid composition of cell membranes may have a strong impact on the cascading cellular responses. Cytokines are one such type of cellular response products that play a key role in the progression or suppression of inflammatory and immune-related processes [8].

Omega-3 fatty acid C18:3n3 was negatively associated with IL-6 at baseline. Additionally, during a 1-year period, changes in this fatty acid were negatively associated with changes in circulating IL-1b levels. These associations are consistent with previous epidemiological studies assessing omega-3 fatty acids in cell membranes [9]. Clinical trials also showed beneficial effects of omega-3 fatty acids on inflammation. For example, a recent placebo-controlled clinical trial conducted for eight weeks in adults in middle to late adulthood showed significant reductions in plasma levels of inflammatory cytokines (IL-6, IL-1b) after supplementation with C20:5n3 and C22:6n3 [10]. Furthermore, dietary supplementation with their precursor, C18:3n3 (alpha-linolenic acid), for 3 months significantly decreased C-reactive protein and IL-6 levels in dyslipidemic patients [11].

Contrary to the established beneficial effects of omega-3 fatty acids on inflammation, findings from studies on omega-6 fatty acids have been inconsistent [12]. In the Kuopio Ischaemic Heart Disease Risk Factor Study, serum total n-6 PUFA and linoleic acid, the predominant n-6 PUFAs, were associated with lower C-reactive protein (CRP) values [13]. Because of multiple potentially competing effects on many metabolic pathways, the effects of n-6 PUFAs on inflammation development are difficult to predict. Our findings are consistent with the prior hypothesis that omega-6 fatty acids may be pro-inflammatory. In our study, the conversion products of C18:2n6 (linoleic acid), which are C20:3n6 (dihomo-gamma-linolenic acid) and C22:5n6 (docosapentaenoic acid) were positively associated with IL-8 at baseline. Furthermore, the 1-year changes in the levels of the end product, C22:5n6, from the pathway of conversion of linoleic acid were positively associated with IL-8 changes. Interestingly, C20:2n6 (known as eicosadienoic acid) was directly associated with the pro-inflammatory cytokine IL-6 at baseline and with the anti-inflammatory IL-10 at 1 year. This fatty acid has at least two metabolic fates in mammalian cells: one is conversion to dihomo-gamma-linolenic acid and arachidonic acid, while the other leads to the formation of sciadonic acid, which has shown anti-inflammatory properties in murine models [14]. It has been suggested that eicosadienoic acid may reduce the pro-inflammatory effects caused by linoleic acid and thereby lower the risk of inflammation [15].

The mechanisms explaining the relationship of omega-3 and omega-6 fatty acids with inflammation are not fully understood. Briefly, omega-3 and omega-6 fatty acids may contribute to inflammation by cell membrane activation, following a series of conversions of omega-3 or omega-6 fatty acids by cyclooxygenases (COXs) and lipoxygenases (LOXs). The conversion products are prostaglandins (PGs), thromboxanes (TXs), and leukotrienes (LTs), based on the fatty acid converted. Depending on the unsaturated fatty acid undergoing the conversion, varied products are formed which either induce inflammation or suppress inflammation. However, the contributions of unsaturated fatty acids in inflammation are not just limited via cell membrane activation, but also by altering the expression of inflammatory genes. Animal and cell culture studies have demonstrated the role of unsaturated fatty acids in the alteration of NF-kB, which in turn controls the transcription of many genes responsible for the production of cytokine, chemokines, and adhesion molecules [16].

Regarding MUFAs, in our study, C16:1n7trans (palmitelaidic acid) was found to be inversely associated with IL-6 at baseline, and with all measured inflammatory markers at 1 year. This observed anti-inflammatory tendency may be related with previous results of higher circulating C16:1n7trans indicating a better metabolic profile from the Multi-Ethnic Study of Atherosclerosis (MESA) [17] and another cohort from the USA [18]. On the other hand, we also observed that the structural variant C16:1n7cis (palmitoleic acid) was positively associated with IL-1b at 1 year. However, this cis MUFA is a lipokine [19] that has been reported to be negatively associated with inflammation and other associated metabolic disorders [20]. This contradictory result needs further investigation. It has been reported that serum levels of the C16:1n7cis form were positively correlated with CRP in men [21]. The role of C16:1n7cis in the pathway of IL-1b and IL-6 needs further investigation [22].

We also observed associations between one n-9 MUFA C20:1n9 (gondoic acid) and its shortening C18:1n9 (oleic acid) and elongation C24:1n9 (nervonic acid) products with inflammatory markers. To our knowledge, our study is the first to demonstrate a direct association between C20:1n9 and some inflammatory markers. We observed a negative association of C20:1n9 with IFN-g and IL-8 at baseline. Our results are in line with those reported in the Diabetes Risk Assessment study [23]. In this study, metabolically unhealthy obese individuals who had higher risk of developing inflammation compared to the metabolically healthy obese individuals showed a significantly lower percentage of C20:1n9 in serum. In our study, nervonic acid was negatively associated with IL-1b at 1 year. Serum nervonic acid levels have been related to serum plasmalogens, which are endogenous antioxidants and could offer protection against chronic inflammation [24], but when measured in erythrocytes they were directly correlated with peripheral IL-6 concentrations (*r* = 0.082, *p* < 0.001) [25].

Most of the literature points towards the negative role of SFAs on health. However, not all SFAs exhibit the same postprandial inflammatory activity. Lee et al. [26] reported different effects of SFAs on the inflammatory gene expression. C12:0 showed the highest activity, whereas C14:0 and C18:0 showed little pro-inflammatory activity. However, in a previous work by Fernandez-Real et al. [27], a positive correlation between serum C14:0 and IL-6 in 232 adults including overweight and lean participants was observed. In our study, C14:0 (myristic acid) and C20:0 (arachidic acid) were negatively associated with IL-6 at baseline. In our study, we found C14:0 to be negatively associated with IL-10 as well pro-inflammatory IL-1b at 1 year. These contradictory results need further investigation in order to understand the role of myristic acid on inflammation. The results obtained with IL-10 at both baseline and 1 year must be interpreted with care, as the role of circulating IL-10 has been shown to be either pro-inflammatory or anti-inflammatory depending on the environment in which it is present [28].

Regarding the lack of inconsistency in findings between baseline and 1 year, this could be due to the differences in sample size (the sample size was reduced to almost half at 1 year). Also, we recognize that even though we adjusted the analyses for intervention groups, we cannot exclude the potential confounding effects of interventions on fatty acids status.

The results of the present study should be interpreted in the context of its limitations and strengths. First, this study is of cross-sectional nature and causality cannot be established. Second, although we adjusted for potential confounders, residual confounders cannot be excluded. Third, participants were elderly Mediterranean individuals at high cardiovascular risk and this may limit the generalizability of the findings to other age groups or populations. Finally, there might be a selection bias due to the nature of the study design (case-control) from which we obtained our study population. Strengths include the objective measurement of twenty-two individual fatty acids in blood cell membranes and five inflammatory markers in serum, representing a wide variety of inflammation pathways. In addition, the assessment of total cell membranes instead of serum circulating levels of fatty acids has the advantage of better representing the long-term fatty acid status [29].

## 5. Conclusions

In conclusion, this study adds to the growing body of evidence suggesting potential differences in inflammatory or anti-inflammatory properties of fatty acids in blood cell membranes in older Mediterranean adults at high CVD risk. The potential mechanisms linking the identified fatty acids and inflammation must be further investigated.

## Figures and Tables

**Table 1:** Baseline characteristics of the study population.

Characteristics	Total
*n*	282
Female sex, (%)	38.8
Age (Years)	67.9 ± 6.6
BMI (kg/m^2^)	29.4 ± 3.1
Waist circumference (cm)	101.0 ± 8.5
Type 2 diabetes, (%)	55.8
Statins use, (%)	65.6
Physical activity, METs/d	288.9 ± 266.0
Dyslipidemia, (%)	68.2
HDL cholesterol (mg/dL)	51.5 ± 13.4
LDL cholesterol (mg/dL)	133.3 ± 38.8
Triglycerides (mg/dL)	148.8 ± 76.0
Smoking %	
Current	19.1
Former	33.9
Never	47.0
Inflammatory markers (pg/mL)	
IFNγ	17.7 ± 10.5
IL-6	3.9 ± 4.3
IL-8	11.6 ± 9.3
IL-10	25.4 ± 30.5
IL-1b	2.3 ± 1.5

BMI, body mass index; WC, waist circumference; MET, metabolic equivalent; HDL, High density lipoprotein; LDL, Low density lipoprotein; IFNγ, interferon-gamma; IL, interleukin.

**Table 2:** Fatty acids percentages (mean ± standard deviation) in blood cell membranes at baseline in the study population.

Fatty Acids	Total (%)
C14:0	1.04 ± 0.52
C16:0	22.48 ± 3.06
C16:1n7trans	0.23 ± 0.11
C16:1n7cis	0.43 ± 0.21
C18:0	18.54 ± 2.1
C18:1n9cis	17.47 ± 2.59
C18:1n9trans	1.45 ± 0.25
C18:2n6	11.16 ± 2.09
C18:3n3	0.1 ± 0.05
C20:0	0.21 ± 0.09
C20:1n9	0.37 ± 0.12
C20:2n6	0.32 ± 0.12
C20:3n6	1.75 ± 0.43
C20:4n6	14.44 ± 2.99
C20:5n3	0.58 ± 0.37
C22:0	0.15 ± 0.16
C22:4n6	2.53 ± 0.81
C22:5n6	0.43 ± 0.18
C22:5n3	1.45 ± 0.44
C24:0	0.3 ± 0.3
C22:6n3	4.4 ± 1.41
C24:1n9	0.3 ± 0.31
Total SFA	42.69 ± 4.52
Total MUFA	18.55 ± 2.69
Total n-6 PUFA	30.6 ± 4.96
LCn-3 PUFA	6.42 ± 2.02
Total n-3 PUFA	6.51 ± 2.03
Omega-3 index	4.98 ± 1.67

SFA, saturated fatty acid; MUFA, monounsaturated fatty acid; PUFA, polyunsaturated fatty acid.

**Table 3:** Fatty acids ranked from the highest to the lowest elastic net positive or negative regression coefficients for inflammatory markers at baseline (*n* = 282).

Inflammatory Markers	Fatty Acids	Coefficient	Fatty Acids	Coefficient
IFNγ			C20:1n9	−0.091
IL-6	C20:2n6	0.022538562	C14:0	−0.077
			C20:0	−0.065
			C16:1n7trans	−0.042
			C18:3n3	−0.014
IL-8	C20:3n6	0.068	Total MUFA	−0.118
			C14:0	−0.117
	C22:5n6	0.048		-
			
IL-10	None	None	None	None
IL–1b	None	None	None	None

Adjusted for age, sex, body mass index, smoking, diabetes, use of statins (yes/no). IFNγ, interferon-gamma; IL, interleukin; MUFA, monounsaturated fatty acid.

**Table 4:** Fatty acids ranked from the highest to the lowest elastic net positive or negative regression coefficients for inflammatory markers at 1 year (*n* = 143).

Inflammatory Markers	Fatty Acids	Coefficient	Fatty Acids	Coefficient
IFNγ			C16:1n7trans	−0.079
			C24:1n9	−0.053
				-
				-
IL-6			C16:1n7trans	−0.099
IL-8	C22:5n6	0.046	C16:1n7trans	−0.189
			C18:1n9cis	−0.079
IL-10	C20:2n6	0.059	C14:0	−0.334
			C16:1n7trans	−0.168
IL-1b	C16:1n7cis	0.093	C14:0	−0.137
			C16:1n7trans	−0.123
			C24:1n9	−0.039
			C18:3n3	−0.018

Adjusted for age, sex, body mass index, smoking, diabetes, use of statins (yes/no), intervention group. IFNγ, interferon-gamma; IL, interleukin.

## Supplementary Materials

The following are available online at https://www.mdpi.com/2072-6643/11/3/576/s1, Table S1: Fatty acids percentages (mean ± standard deviation) in blood cell membranes at baseline in the study population, Table S2: Inflammatory markers concentrations (pg/ml) (mean ± standard deviation) in serum at baseline in the study population, Table S3: Spearman’s correlation analysis between baseline and 1-year levels of fatty acids, Table S4: Spearman’s correlation analysis between baseline and 1-year levels of inflammatory markers.

## References

[1] Armstrong E., Morrow D., Sabatine M. (2006). Inflammatory Biomarkers in Acute Coronary Syndromes. Circulation.

[2] Calder P.C. (2017). Omega-3 fatty acids and inflammatory processes: From molecules to man. Biochem. Soc. Trans..

[3] Bersch-Ferreira A.C., Sampaio G.R., Gehringer M.O., da Silva Torres E.A.F., Ross-Fernandes M.B., da Silva J.T., Torreglosa C.R., Kovacs C., Alves R., Magnoni C.D. (2018). Association between plasma fatty acids and inflammatory markers in patients with and without insulin resistance and in secondary prevention of cardiovascular disease, a cross-sectional study. Nutr. J..

[4] Estruch R., Ros E., Salas-Salvadó J., Covas M.-I., Corella D., Arós F., Gómez-Gracia E., Ruiz-Gutiérrez V., Fiol M., Lapetra J. (2018). Primary Prevention of Cardiovascular Disease with a Mediterranean Diet Supplemented with Extra-Virgin Olive Oil or Nuts. N. Engl. J. Med..

[5] Sala-Vila A., Harris W.S., Cofán M., Pérez-Heras A.M., Pintó X., Lamuela-Raventós R.M., Covas M.I., Estruch R., Ros E. (2011). Determinants of the omega-3 index in a Mediterranean population at increased risk for CHD. Br. J. Nutr..

[6] Elosua R., Marrugat J., Molina L., Pons S., Pujol E. (1994). Validation of the Minnesota Leisure Time Physical Activity Questionnaire in Spanish men. The MARATHOM Investigators. Am. J. Epidemiol..

[7] Friedman J., Hastie T., Tibshirani R. (2010). Regularization Paths for Generalized Linear Models via Coordinate Descent. J. Stat. Softw..

[8] Lin W.-W., Karin M. (2007). A cytokine-mediated link between innate immunity, inflammation, and cancer. J. Clin. Investig..

[9] Ferrucci L., Cherubini A., Bandinelli S., Bartali B., Corsi A., Lauretani F., Martin A., Andres-Lacueva C., Senin U., Guralnik J.M. (2006). Relationship of Plasma Polyunsaturated Fatty Acids to Circulating Inflammatory Markers. J. Clin. Endocrinol. Metab..

[10] Tan A., Sullenbarger B., Prakash R., McDaniel J.C. (2018). Supplementation with eicosapentaenoic acid and docosahexaenoic acid reduces high levels of circulating proinflammatory cytokines in aging adults: A randomized, controlled study. Prostaglandins Leukot. Essent. Fat. Acids.

[11] Rallidis L.S., Paschos G., Liakos G.K., Velissaridou A.H., Anastasiadis G., Zampelas A. (2003). Dietary alpha-linolenic acid decreases C-reactive protein, serum amyloid A and interleukin-6 in dyslipidaemic patients. Atherosclerosis.

[12] Innes J.K., Calder P.C. (2018). Omega-6 fatty acids and inflammation. Prostaglandins Leukot. Essent. Fat. Acids.

[13] Virtanen J.K., Mursu J., Voutilainen S., Tuomainen T.-P. (2018). The associations of serum n-6 polyunsaturated fatty acids with serum C-reactive protein in men: The Kuopio Ischaemic Heart Disease Risk Factor Study. Eur. J. Clin. Nutr..

[14] Chen S.-J., Huang W.-C., Yang T.-T., Lu J.-H., Chuang L.-T. (2012). Incorporation of sciadonic acid into cellular phospholipids reduces pro-inflammatory mediators in murine macrophages through NF-κB and MAPK signaling pathways. Food Chem. Toxicol..

[15] Huang Y.-S., Huang W.-C., Li C.-W., Chuang L.-T. (2011). Eicosadienoic acid differentially modulates production of pro-inflammatory modulators in murine macrophages. Mol. Cell. Biochem..

[16] Wan J.B., Huang L.L., Rong R., Tan R., Wang J., Kang J.X. (2010). Endogenously Decreasing Tissue n-6/n-3 Fatty Acid Ratio Reduces Atherosclerotic Lesions in Apolipoprotein E–Deficient Mice by Inhibiting Systemic and Vascular Inflammation. Arterioscler. Thromb. Vasc. Biol..

[17] Mozaffarian D., de Oliveira Otto M.C., Lemaitre R.N., Fretts A.M., Hotamisligil G., Tsai M.Y., Siscovick D.S., Nettleton J.A. (2013). Trans-Palmitoleic acid, other dairy fat biomarkers, and incident diabetes: The Multi-Ethnic Study of Atherosclerosis (MESA). Am. J. Clin. Nutr..

[18] Mozaffarian D., Cao H., King I.B., Lemaitre R.N., Song X., Siscovick D.S., Hotamisligil G.S. (2010). Trans-palmitoleic acid, metabolic risk factors, and new-onset diabetes in U.S. adults: A cohort study. Ann. Intern. Med..

[19] Cao H., Gerhold K., Mayers J.R., Wiest M.M., Watkins S.M., Hotamisligil G.S. (2008). Identification of a lipokine, a lipid hormone linking adipose tissue to systemic metabolism. Cell.

[20] Souza C.O., Vannice G.K., Neto J.C.R., Calder P.C. (2017). Is Palmitoleic Acid a Plausible Nonpharmacological Strategy to Prevent or Control Chronic Metabolic and Inflammatory Disorders?. Mol. Nutr. Food Res..

[21] Petersson H., Basu S., Cederholm T., Riserus U. (2008). Serum fatty acid composition and indices of stearoyl-CoA desaturase activity are associated with systemic inflammation: Longitudinal analyses in middle-aged men. Br. J. Nutr..

[22] Djoussé L., Weir N., Hanson N., Tsai M., Gaziano J. (2012). Plasma Phospholipid Concentration of Cis-Palmitoleic Acid and Risk of Heart Failure. Circ. Hear. Fail..

[23] Perreault M., Zulyniak M.A., Badoud F., Stephenson S., Badawi A., Buchholz A., Mutch D.M. (2014). A Distinct Fatty Acid Profile Underlies the Reduced Inflammatory State of Metabolically Healthy Obese Individuals. PLoS ONE.

[24] Yamazaki Y., Kondo K., Maeba R., Nishimukai M., Nezu T., Hara H. (2014). The Proportion of Nervonic Acid in Serum Lipids is Associated with Serum Plasmalogen Levels and Metabolic Syndrome. J. Oleo Sci..

[25] Delgado G.E., Kramer B.K., Lorkowski S., Marz W., von Schacky C., Kleber M.E. (2017). Individual omega-9 monounsaturated fatty acids and mortality-The Ludwigshafen Risk and Cardiovascular Health Study. J. Clin. Lipidol..

[26] Lee J.Y., Sohn K.H., Rhee S.H., Hwang D. (2001). Saturated fatty acids, but not unsaturated fatty acids, induce the expression of cyclooxygenase-2 mediated through Toll-like receptor 4. J. Biol. Chem..

[27] Fernández-Real J.-M., Broch M., Vendrell J., Ricart W. (2003). Insulin Resistance, Inflammation, and Serum Fatty Acid Composition. Diabetes Care.

[28] Welsh P., Murray H.M., Ford I., Trompet S., de Craen A.J., Jukema J.W., Stott D.J., McInnes I.B., Packard C.J., Westendorp R.G. (2011). Circulating interleukin-10 and risk of cardiovascular events: A prospective study in the elderly at risk. Arterioscler. Thromb. Vasc. Biol..

[29] Arab L. (2003). Biomarkers of Fat and Fatty Acid Intake. J. Nutr..

