# Supplementary material for: Fatty Acids Composition of Blood Cell Membranes and Peripheral Inflammation in the PREDIMED Study: A Cross-Sectional Analysis
Source: Nutrients. 2019 Mar 7;11(3):576. doi: 10.3390/nu11030576 (PMC6471987; doi:10.3390/nu11030576)
Supplement: Supplementary file 1 [file nutrients-11-00576-s001.pdf]

Supplementary Table S1. Fatty acids percentages (mean ± standard deviation) in blood cell membranes at baseline in the study population

Fatty acids	Total	Control (n=177)	Cases (n=105)
C14:0	1.04 ± 0.52	1.07 ± 0.60*	0.95 ± 0.30
C16:0	22.48 ± 3.06	22.35 ± 2.98	22.68 ± 3.18
C16:1n7trans	0.23 ± 0.11	0.21 ± 0.11	0.23 ± 0.09
C16:1n7cis	0.43 ± 0.21	0.40 ± 0.19	0.45 ± 0.22
C18:0	18.54 ± 2.1	18.46 ± 2.18	18.64 ± 1.96
C18:1n9cis	17.47 ± 2.59	17.11 ± 2.40	18.06 ± 2.78
C18:1n9trans	1.45 ± 0.25	1.41 ± 0.26	1.49 ± 0.21
C18:2n6	11.16 ± 2.09	11.24 ± 2.03	11.00 ± 2.17
C18:3n3	0.1 ± 0.05	0.09 ± 0.04	0.09 ± 0.04
C20:0	0.21 ± 0.09	0.19 ± 0.08	0.20 ± 0.08
C20:1n9	0.37 ± 0.12	0.33 ± 0.10	0.40 ± 0.11
C20:2n6	0.32 ± 0.12	0.31 ± 0.13	0.31 ± 0.07
C20:3n6	1.75 ± 0.43	1.72 ± 0.41	1.76 ± 0.44
C20:4n6	14.44 ± 2.99	14.59 ± 2.88	14.14 ± 3.16
C20:5n3	0.58 ± 0.37	0.57 ± 0.40	0.57 ± 0.30
C22:0	0.15 ± 0.16	0.17 ± 0.16*	0.10 ± 0.10
C22:4n6	2.53 ± 0.81	2.62 ± 0.81*	2.35 ± 0.74
C22:5n6	0.43 ± 0.18	0.44 ± 0.18*	0.38 ± 0.14
C22:5n3	1.45 ± 0.44	1.48 ± 0.45*	1.38 ± 0.40
C24:0	0.3 ± 0.3	0.34 ± 0.32	0.20 ± 0.21
C22:6n3	4.4 ± 1.41	4.43 ± 1.45*	4.30 ± 1.32
C24:1n9	0.3 ± 0.31	0.33 ± 0.33	0.21 ± 0.24
Total SFA	42.69 ± 4.52	42.61 ± 4.49	42.80 ± 4.60
Total MUFA	18.55 ± 2.69	18.20 ± 2.47	19.13 ± 2.93
Total n-6 PUFA	30.6 ± 4.96	30.95 ± 4.74	29.96 ± 5.26
LCn-3 PUFA	6.42 ± 2.02	6.49 ± 2.10	6.26 ± 1.88
Total n-3 PUFA	6.51 ± 2.03	6.59 ± 2.10	6.36 ± 1.88
Omega-3 index	4.98 ± 1.67	5.01 ± 1.73	4.88 ± 1.53

Student's t-test was used for comparison of fatty acid levels in cases and controls. *p value<0.05. SFA, saturated fatty acid; MUFA, monounsaturated fatty acid; PUFA, polyunsaturated fatty acid; LC, long-chain.

Supplementary Table S2. Inflammatory markers concentrations (pg/ml) (mean ± standard deviation) in serum at baseline in the study population

Inflammatory markers	Control	Cases
IFNg	18.172 ± 11.536	16.797 ± 8.791
IL10	25.752 ± 36.138	22.524 ± 16.418
IL1b	2.209 ± 1.454	2.241 ± 1.71
IL6	3.956 ± 4.768	3.78 ± 3.321
IL8	11.025 ± 8.151	12.691 ± 10.941

Student's t-test was used for comparison of inflammatory markers levels in cases and controls. No significant differences were found ($p>0.05$).

Supplementary Table S3. Spearman's correlation analysis between baseline and 1-year levels of fatty acids

**Fatty acids**	**Spearman r**	**p value**
C14:0	0.77	<0.001
C16:0	0.58	<0.001
C16:1n7cis	0.64	<0.001
C16:1n7trans	0.61	<0.001
C18:0	0.52	<0.001
C18:1n9cis	0.69	<0.001
C18:1n9trans	0.68	<0.001
C18:2n6	0.69	<0.001
C18:3n3	0.28	<0.001
C20:0	0.45	<0.001
C20:1n9	0.52	<0.001
C20:2n6	0.45	<0.001
C20:3n6	0.57	<0.001
C20:4n6	0.51	<0.001
C20:5n3	0.63	<0.001
C22:0	0.85	<0.001
C22:4n6	0.74	<0.001
C22:5n6	0.73	<0.001
C22:5n3	0.55	<0.001
C22:6n3	0.55	<0.001
C24:0	0.85	<0.001
C24:1n9	0.84	<0.001

Supplementary Table S4. Spearman's correlation analysis between baseline and 1-year levels of inflammatory markers

**Inflammatory markers**	**Spearman r**	**p value**
IFN-g	0.85	<0.001
IL-6	0.68	<0.001
IL-8	0.64	<0.001
IL-10	0.83	<0.001
IL-1b	0.86	<0.001
